# Effectiveness of nursing care intervention for alleviation of anxiety, pain and functional improvement amongst patients undergoing ambulatory surgery: A systematic review and meta-analysis

**DOI:** 10.12669/pjms.40.6.9472

**Published:** 2024-07

**Authors:** Hongna Xu, Yan Shi

**Affiliations:** 1 Hongna Xu, Tongji University School of Medicine, Shanghai 200092, China. Deparment of Hepatobitiary Surgery, Ningbo Yinzhou No.2 Hospital, Ningbo, Zhejiang Province 315100, China; 2 Yan Shi Department of Nursing, Tenth People’s Hospital of Tongji University, Shanghai 200072, China

**Keywords:** Ambulatory Surgical Procedures, Meta-Analysis, Nursing, Pain

## Abstract

**Background & Objective:**

Ambulatory surgeries are increasingly prevalent, yet they often result in postoperative pain and anxiety, impacting patient recovery and satisfaction. Effective management of these complications is crucial, and nursing care interventions have been proposed as a potential solution. This meta-analysis aims to evaluate the effectiveness of nursing care interventions in reducing pain and anxiety and improving functional status among patients undergoing ambulatory surgery.

**Methods:**

A comprehensive literature search done on December 2023 of PubMed Central, MEDLINE, Scopus, Google Scholar, Cochrane library, CINAHL, and trial registries was done for studies from inception till November 2023, that met predefined eligibility criteria. Standardized mean differences (SMD) for continuous outcomes and odds ratios (OR) for binary outcomes were calculated using a random-effects inverse-variance model. Sensitivity analysis was performed to assess the robustness of the findings, and heterogeneity was evaluated using I² statistics.

**Results:**

Nine studies were included. Pooled analysis revealed a significant reduction in pain (SMD = -1.224, 95% CI: -2.445 to -0.003, p=0.049) and anxiety (SMD = -1.53, 95% CI: -2.77 to -0.28, p=0.016) among patients receiving nursing care interventions, with substantial heterogeneity (I² = 98.2% for pain and 96.6% for anxiety). However, no significant improvement was observed in the functional status (SMD = -0.28, 95% CI: -0.35 to 0.91, p=0.385). Sensitivity analysis confirmed the stability of these results.

**Conclusion:**

Nursing care interventions are effective in significantly reducing pain and anxiety in patients undergoing ambulatory surgery. However, their impact on improving functional status remains inconclusive. Our findings underscore the importance of integrating nursing care into postoperative management protocols in ambulatory surgeries and highlight areas for future research, particularly concerning functional recovery.

## INTRODUCTION

In recent years, the landscape of surgical procedures has evolved significantly, with ambulatory surgery becoming increasingly prevalent. This shift towards outpatient surgical settings is driven by advances in medical technology, anaesthesia, and a focus on cost-effectiveness and patient-centred care.^1^ Nevertheless, patients undergoing outpatient surgery frequently encounter postoperative challenges, such as anxiety and pain 2,3 which are not only detrimental to the immediate recovery but can also have lasting impacts on the overall health and well-being of the patients.

The management of anxiety and pain in patients undergoing ambulatory surgery is a multifaceted challenge that requires a comprehensive approach, since unmanaged or poorly managed pain and anxiety can lead to prolonged recovery times, increased incidence of postoperative complications, higher readmission rates, and overall reduced patient satisfaction.^2^-^4^ Nurses are often the primary caregivers who monitor, assess, and manage the pain and anxiety experienced by patients, and their interventions can range from pharmacological management to psychological support.^5^-^8^

Numerous studies focused on pain and anxiety management in surgical patients.^9^-^11^ While there is an understanding that pain is not merely a physical sensation but is also influenced by psychological and social factors,^12^ there is a noticeable gap in comprehensive analyses, particularly in the context of ambulatory surgery.

This meta-analysis aims to summarize the existing data and to evaluate the effectiveness of various nursing care interventions in managing anxiety, pain and functional status in patients undergoing ambulatory surgery.

## METHODS

PubMed Central, MEDLINE, Scopus, Google Scholar, Cochrane library, CINAHL, trial registries were searched for relevant studies from inception till November 2023, with no language restrictions. Additionally, reference list of retrieved studies was manually searched.

### Search terms:

“Daycare Surgery”, “Ambulatory Surgery”, “Outpatient Surgery”, “Nurses”, “Nursing”, “Randomized Controlled Trial”, “Nursing Care” along with Boolean Operators “AND”, “OR” and “NOT”

### Study selection:

Two researchers independently conducted literature searches, screening titles, abstracts, and keywords. Abstracts and full texts of relevant papers were further screened to select studies meeting the review’s inclusion criteria. Any selection disagreements were resolved through consensus. The review was reported using the PRISMA checklist, ensuring adherence to established standards.^13^ The study was registered at PROSPERO, No. CRD42023494270.

### Inclusion criteria:

- Studies performed in adult participants undergoing day (ambulatory, outpatient) surgery which does not require overnight stay postoperatively.
- Studies reporting nursing care intervention i.e., nurses should be the primary provider of intervention in the form of education, or applying anxiety alleviation techniques like motivational interviewing, counselling etc.
- Studies comparing the nursing care intervention with standard care.
- Randomized controlled trials (RCTs) or non-RCTs or quasi-experimental trials;
- Studies describing patient-reported outcomes like pain, anxiety or functional status (ability to perform usual daily activities).

### Exclusion criteria:

- Case reports, series, unpublished literature.
- Studies conducted in children.
- *Top of Form*
- *Bottom of Form*

### Data collection:

The primary investigator meticulously gathered key characteristics, such as extraction date, study title, and authors, study design, participant demographics, and setting, participant count in each arm, baseline and endline measures, and inclusion/exclusion criteria, specifics of intervention and comparison groups, and follow-up duration, primary and secondary outcomes, timing of assessments, and other quality-assessment details. The primary investigator systematically compiled this data, and the accuracy was further verified by discussion of the investigators.

### Risk of bias assessment:

Two investigators were responsible for assessing the quality of the included studies using the Cochrane risk of bias (RoB-2) tool for RCTs^14^ and risk of bias in non-randomized studies (ROBINS-I) tool^15^ for non-RCTs. Based on the various parameters within these tools, risk of bias was graded as low, high or some concerns.

### Statistical analysis:

STATA version 14.2. was used for analysis. For continuous outcomes, means, standard deviations, and sample sizes from both groups were used to calculate the standardized mean difference (SMD) with a 95% confidence interval (CI). Binary outcomes were analysed using event frequencies in both arms, deriving odds ratios (OR) with a 95% CI. The random-effects model, employing the inverse variance method, was used.^16^ Assessment of heterogeneity was done through chi-square and I^2^ statistics.^16^ Sensitivity analysis identified the impact of individual studies on overall estimates. P<0.05 was considered statistically significant.

## RESULTS

**Fig.1:**
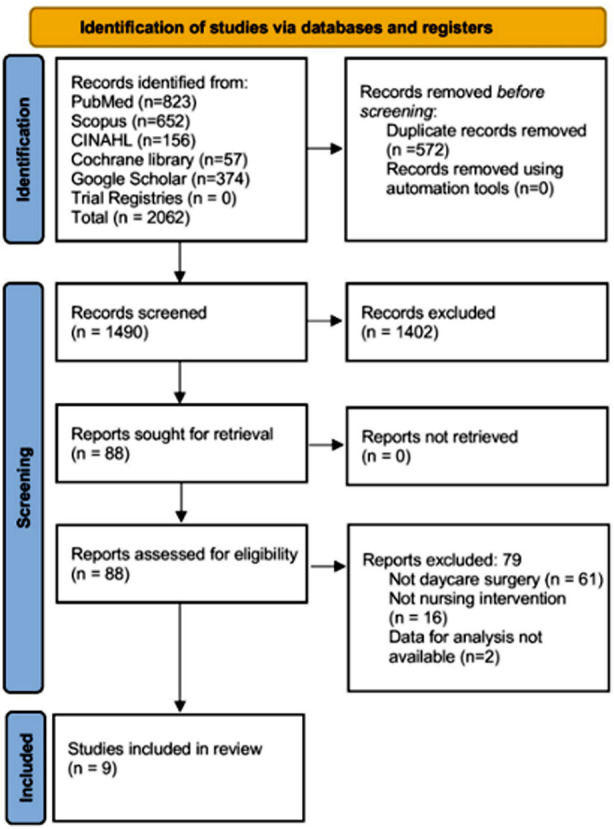
PRISMA flowchart.

A total of 2,062 records were identified by the search across five databases. After screening and assessing for eligibility, 79 reports were excluded for not meeting the criteria related to day care surgery, nursing intervention specificity, or data availability. Nine studies were included in the analysis (Fig.1).^9^–^11^,^17^–^22^

### Characteristics of the included studies:

As summarized in Table-I, the included studies encompassed a diverse range of participants, interventions, and settings. The sample sizes varied from 40 to 292 participants per study, with a total of 1668 patients. The mean age of participants across studies ranged from 42.2 to 61 years. Nursing interventions varied from attentional focus and symptom management to predictive nursing and preoperative education. In terms of the risk of bias (Table-IIA & B), three studies were rated high, two had some concerns, and four were considered low risk of bias.

**Table-I:** Characteristics of the included studies (N=9).

Author and Year	Study design	Country	Study participants	Sample size	Intervention details	Anxiety scale	Pain scale	Timing of intervention	Timing of pain and anxiety assessment	Mean age in years
Allard 2006^11^	RCT	Canada	Breast cancer patients who were outpatients and undergoing surgery as part of the initial treatment for their cancer	I=61 C=56	Attentional focus and Symptom Management Intervention (AFSMI) provided by nurses	Profile of Mood subscale for anxiety	NA	3-4 days and 10-11 days after surgery	3 different occasions during follow-up with endline at 17-18 days after surgery	53.6
Brand 2013^21^	Quasi experimental	USA	Adults patients undergoing day care surgery	I=45 C=41	Hand massage procedure by the nurses	Visual analog scale for anxiety	NA	Part of preoperative care in ambulatory surgery	Immediately before participants were transported to operating room	57.8
Calafell 2011^18^	Quasi experimental	Spain	Patients undergoing hernia surgery as day care procedure	I=292 C=205	Educational intervention in graphic form	NA	Visual analog scale	Presurgical nursing care	Within 24 hours postoperatively	52.8
Jones 2011^20^	RCT	USA	Patients undergoing ambulatory arthroscopic surgery	I=52 C=50	Nurse coached telephonic educational intervention	NA	NA	On the day of surgery	72 hours and 1 week postoperatively	45.9
Lulu 2023^10^	Non-RCT	China	Patients undergoing ambulatory anorectal surgery	I=40 C=40	Predictive nursing intervention	Hamilton anxiety scale	Visual analog scale	Time of surgery and throughout postoperative period	NR	42.2
Pereira 2016^19^	RCT	Portugal	Patients undergoing general ambulatory surgery	I=52 C=52	Nurse delivered personalized intervention through empathic patient centered interview	State trait Anxiety Inventory	Numerical rating scale	Immediate preoperative care	Immediately after intervention and one month after surgery	43.7
Porras-Gonzalez 2014^17^	Quasi experimental	Spain	Patients undergoing major ambulatory surgery	I=185 C=195	Preoperative nurse delivered educational sessions	NA	Visual analog scale	Before anesthetist’s assessment and after inclusion on waiting list for surgery	Following surgery and before discharge	NR
Sawhney 2017^9^	RCT	Canada	Patients undergoing ambulatory inguinal hernia repair	I=40 C=42	Preoperative nurse delivered educational intervention	NA	Numerical rating scale	Immediately following usual care which is maximum of one week before surgery	Day 2 postoperatively	61
Valeberg 2021^22^	RCT	Norway	Patients undergoing ambulatory shoulder surgery	I=101 C=119	Preoperative nurse delivered psycho-educational intervention	NA	Numerical rating scale	Postoperative day 2,3 and 7	7 days after surgery	51

C – Control; I – Intervention; NR – Not reported; NA – Not applicable; RCT – randomized controlled trial; USA – United States of America.

**Table IIA:** Risk of bias assessment for RCTs (n=5).

Study No.	Author and year	Randomization process	Deviation from intended intervention	Missing outcome data	Measurement of the outcome	Selection of the reported results	Overall
1.	Allard 2006^11^	Low	Low	High	High	High	High
2.	Jones 2011^20^	Some concerns	Some concerns	High	Low	High	High
3.	Pereira 2016^19^	Some concerns	Some concerns	Low	Low	Low	Some concerns
4.	Sawhney 2017^9^	Low	Low	High	Low	Some concerns	High
5.	Valeberg 2021^22^	Some concerns	Some concerns	High	Low	High	High

**Table IIB:** Risk of bias assessment for non-RCTs (n=4).

Study No	Author and year	Bias due to confounding	Bias in selection of participants	Bias in classification of interventions	Bias due to deviations from intended interventions	Bias due to missing data	Bias in measurement of outcome	Bias in selection of reported result	Overall
1.	Brand 2013^21^	Low	Low	High	High	High	High	Some concerns	High
2.	Calafell 2011^18^	Low	Low	Low	Low	Low	Low	Low	Low
3.	Lulu 2023^10^	Low	Low	Low	Low	Some concerns	Low	Some concerns	Some concerns
4.	Porras-Gonzalez 2014^17^	Low	Low	Low	Low	Low	Low	Low	Low

### Effect of nursing care intervention on patient-reported outcomes:

### Pain:

**Fig.2:**
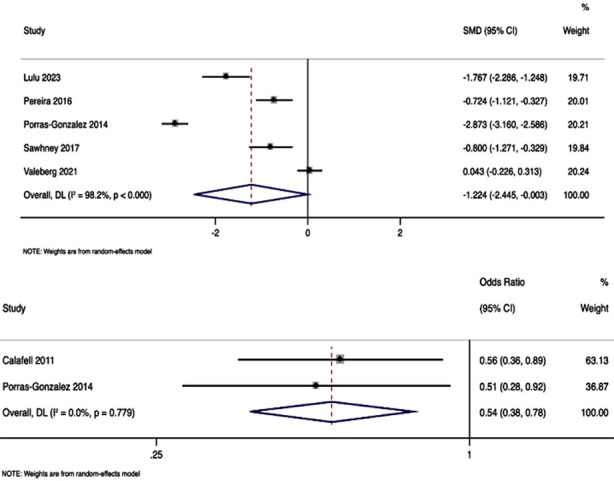
Forest plot showing the effectiveness of nursing care interventions for alleviation of pain in ambulatory surgery patients. A) pain as numerical score B) pain as categorical variable (VAS >3)

Pain was reported in five studies with a total of 852 participants. Pooled SMD was -1.224, with 95%CI of -2.445 to -0.003, and p-value of 0.049, indicating statistically significant reduction in pain score due to nursing care intervention (Fig.2A). The analysis exhibited substantial heterogeneity, as reflected by an I² statistic of 98.2%, suggesting considerable variability among the included studies. Studies have also reported the difference in terms of patients reporting pain score (visual analog scale [VAS] score > 3) as dichotomous variable. This analysis revealed a pooled OR of 0.54 (95%CI: 0.38 to 0.78; p=0.001) with I² statistic of 0% (Fig.2B). This indicates that ambulatory surgery patients receiving nursing care intervention had substantially lower odds of having VAS score > 3 compared to patients in standard care.

### Anxiety:

**Fig.3:**
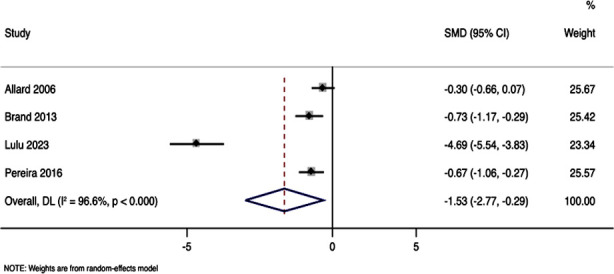
Forest plot showing the effectiveness of nursing care interventions for alleviation of anxiety in ambulatory surgery patients.

Anxiety was assessed in four studies with 387 participants. Pooled SMD was -1.53, with 95%CI of -2.77 to -0.28, and p-value of 0.016, indicating statistically significant reduction in anxiety score in patients who got nursing care intervention (Fig.3). The analysis exhibited substantial heterogeneity (I² = 96.6%) among the included studies.

### Functional status:

**Fig.4:**
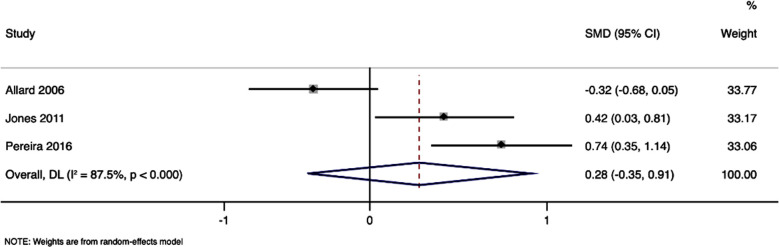
Forest plot showing the effectiveness of nursing care interventions for improvement of functional status in ambulatory surgery patients.

Pooled analysis of three studies, reporting data of the functional status, with 323 participants, showed SMD of -0.28, with 95%CI of -0.35 to 0.91, and p-value of 0.385, indicating no statistical significance (Fig.4). The analysis exhibited substantial heterogeneity, with I² statistic of 87.5%, suggesting substantial variability between the studies.

### Sensitivity analysis:

Sensitivity or leave one out analysis revealed no substantial variations in the magnitude or direction of any outcomes, indicating the absence of single-study effects on the results.

## DISCUSSION

The current meta-analysis critically evaluated the impact of nursing care interventions on patient-reported outcomes in ambulatory surgery settings. Our findings revealed significant reductions in both pain and anxiety levels in patients receiving these interventions, while no statistically significant change was observed in functional status.

Our analysis indicated a substantial reduction in pain scores (SMD = -1.224), a result corroborated by the lower rates of patients reporting higher pain scores (VAS > 3) in the intervention group. Similarly, anxiety levels showed a significant decrease (SMD = -1.53), associated with the nursing care interventions. These results align with the growing body of evidence supporting the efficacy of nursing interventions in managing postoperative discomfort and stress for various types of surgeries.^23^,^24^

The observed effects of nursing interventions on pain and anxiety are consistent with earlier reports emphasizing the role of personalized nursing care in postoperative recovery, such as studies by Pereira et al.^19^ and Sawhney et al.^9^ However, we further extend this knowledge by aggregating data across multiple studies, thereby providing a more comprehensive understanding. The divergence in findings related to functional status highlights the complexity of postoperative recovery, indicating that factors beyond pain and anxiety might influence this outcome.

The significant reductions in pain and anxiety can be attributed to several factors inherent in nursing care. These include personalized attention, effective communication, and the implementation of evidence-based pain management strategies.^25^ The biopsychosocial model of care, which addresses the interplay between physical, psychological, and social factors, is likely instrumental in these outcomes.^26^,^27^ The efficacy of such interventions underscores the vital role of nursing staff in the postoperative recovery process, beyond traditional medical management.

However, the lack of significant improvement in functional status post-intervention warrants a critical examination of our findings in relation to existing literature.^17^-^22^ Studies have suggested that while immediate postoperative outcomes, such as pain and anxiety, can be effectively managed through nursing interventions,^23^-^27^ the long-term recovery of functional status may require a multidisciplinary approach that extends beyond the scope of nursing care alone.^28^,^29^ This includes physical therapy and sustained patient education, emphasizing the complexity of postoperative recovery and the multifactorial nature of functional improvement status.^30^

A major strength of our study lies in its comprehensive approach, and the use of meta-analysis to synthesize data from different sources. The sensitivity analysis further strengthens our findings by confirming their robustness.

### Limitations:

Nevertheless, our study has limitations. High heterogeneity indicates variability in study designs, interventions, and participant characteristics. Additionally, limited number of studies included in some analyses might affect the generalizability of our findings.

Our findings have significant implications for nursing practice. They reinforce the importance of nursing care interventions in managing pain and anxiety post-surgery, suggesting that such practices should be integrated into standard postoperative care protocols.

Furthermore, the results underline the need for ongoing research to optimize nursing interventions, particularly in enhancing postoperative functional recovery. Future research should also focus on exploring the specific components of nursing interventions that most effectively contribute to improved outcomes. Studies examining the impact of these interventions on different types of surgeries and patient demographics are also warranted.

## CONCLUSION

This meta-analysis demonstrates that nursing care interventions significantly reduce pain and anxiety in ambulatory surgery patients, underscoring the importance of such interventions in postoperative care. However, the impact on functional status remains unclear, indicating a need for further research in this area. Our findings highlight the critical role of nursing in enhancing patient outcomes and inform future practices and studies in surgical care.

### Authors’ contributions:

**HX:** Conceived and designed the study.

**HX and YS:** Collected the data and performed the analysis.

**HX:** Was involved in the writing of the manuscript and is responsible for the integrity of the study.

All authors have read and approved the final manuscript.
